# High-speed quantitative X-ray multi-contrast imaging with deep learning based modulated pattern analysis

**DOI:** 10.1107/S1600577526000846

**Published:** 2026-02-17

**Authors:** Zhi Qiao, Yudong Yao, Hongyu Chen, Guohao Du, Pingping Wen, Zhihao Guan, Yajun Tong, Xianbo Shi, Huaidong Jiang

**Affiliations:** a Center of Transformative Science ShanghaiTech University Shanghai 201210 China; b Shanghai Advanced Research Institute Chinese Academy of Sciences Shanghai201210 China; c Advanced Photon Source Argonne National Laboratory Lemont IL60439 USA; d School of Physical Science and Technology ShanghaiTech University Shanghai China; ESRF – The European Synchrotron, France

**Keywords:** X-ray at-wavelength metrology, wavefront sensing, phase contrast imaging, deep learning, speckle tracking

## Abstract

The advent of X-ray multi-contrast imaging methods, providing absorption, phase, and dark-field images, holds tremendous promise for complementary and non-destructive visualization of inner structures within materials and bio-samples. However, the low efficiency in measuring and analyzing X-ray modulated patterns has hindered their application in high-resolution *in situ* imaging. In this work, the Enhanced Scanning Pattern-based Imaging Neural Network (ESPINNet) is introduced as a powerful tool for achieving high-speed, high-resolution quantitative imaging. ESPINNet is faster than correlation-based speckle tracking methods such as XSVT and UMPA, and provides a balanced performance in terms of resolution and speed for data collection by using fewer scanning images. In comparison with our previously developed neural network, ESPINNet introduces the capability to generate dark-field images, further enhancing its versatility. By leveraging scanning patterns, ESPINNet significantly improves resolution and measurement precision. Furthermore, its adaptability to various modulation patterns, including those produced by sandpaper, coded masks, or gratings, ensures broad applicability. These features enable real-time 2D and 3D multi-contrast imaging, positioning ESPINNet as a transformative solution for applications in materials science and biomedical research, particularly for high-speed and *in situ* measurements.

## Abstract 2

This work presents a neural-network framework that accelerates X-ray multi-contrast imaging—simultaneously delivering absorption, phase, and dark-field, while maintaining high spatial resolution, and outperforms correlation-based methods in speed, achieving a favorable balance between resolution and throughput. The method is agnostic to the modulation source (sandpaper, coded masks, gratings), enabling flexible deployment across setups and supporting real-time 2D/3D quantitative imaging for high-speed, *in situ* studies in materials science and biomedical applications.

## 1. Introduction

X-ray imaging has long been a fundamental technique for investigating the internal structures of materials owing to the high penetration depth of X-rays. It has been widely applied across diverse fields, including materials science, medicine, chemistry, and industrial applications (Maire & Withers, 2014 ▸; Sakellariou *et al.*, 2004 ▸; Haddad *et al.*, 1994 ▸; Hampel, 2022 ▸; Seichter *et al.*, 2021 ▸; Wood, 2018 ▸; Kalender, 2006 ▸; Hiriyannaiah, 1997 ▸). Compared with attenuation-based X-ray imaging, quantitative X-ray phase contrast imaging (XPCI) stands out for its heightened sensitivity to light materials such as bio-samples, making it an increasingly valuable tool for exploring the intricate inner structures of bulk materials. Since imaging detectors can only acquire intensity information, various methods have been developed to retrieve phase information from the intensity distribution (Momose, 1995 ▸; Mayo *et al.*, 2003 ▸; Momose *et al.*, 1996 ▸), including propagation-based methods, grating interferometry, speckle-based imaging (SBI) methods, diffraction-based methods and implicit speckle tracking methods proposed recently (Tao *et al.*, 2021 ▸; Bravin *et al.*, 2013 ▸; Beltran *et al.*, 2023 ▸; Morgan & Paganin, 2019 ▸; Paganin *et al.*, 2018 ▸; Pavlov *et al.*, 2020*a* ▸; Pavlov *et al.*, 2020*b* ▸; De Marco *et al.*, 2023 ▸; Celestre *et al.*, 2025 ▸).

Among the aforementioned methods, while the propagation-based method is notable for its simplicity, it lacks the crucial ability for quantitative measurements (Pavlov *et al.*, 2020*a* ▸; Paganin *et al.*, 2018 ▸; Paganin & Morgan, 2019 ▸). Diffraction-based methods, such as coherent diffraction imaging and ptychography, have gained widespread attention for their high resolution and superior imaging quality (Shi *et al.*, 2019 ▸; Wakonig *et al.*, 2019 ▸; Pfeiffer, 2018 ▸). However, diffraction-based imaging methods rely on X-ray photon-counting detectors and high-coherent light sources, which restricts their applications. In contrast, modulated pattern analysis methods, such as speckle-based imaging (SBI) and Talbot grating interferometry, excel in retrieving quantitative information and performing multi-contrast measurements. These methods can proficiently reconstruct phase, attenuation, and dark-field images, enabling comprehensive sample characterization (Wang *et al.*, 2015 ▸; Vittoria *et al.*, 2017 ▸). In addition, the speckle-based imaging method offers significant advantages in flexibility and X-ray source compatibility. It eliminates the need for specific speckle-detector distances or stringent X-ray source requirements and can be applied to low-coherence X-ray sources, such as laboratory-based X-ray systems (Berujon *et al.*, 2012 ▸; Berujon *et al.*, 2015 ▸; Wang *et al.*, 2016 ▸).

To extract multi-contrast information, various SBI methods have been developed, including X-ray speckle tracking (XST), X-ray speckle vector tracking (XSVT), implicit speckle tracking, and unified modulated pattern analysis (UMPA) tailored for both speckle and grating pattern analysis. These methods can be categorized into single-image and scanning modes based on the measurement procedure (Berujon *et al.*, 2020*a* ▸; Berujon *et al.*, 2020*b* ▸; Zdora *et al.*, 2017 ▸; Zdora *et al.*, 2020*a* ▸; Zdora *et al.*, 2020*b* ▸). Single-image measurements prioritize speed over spatial resolution, whereas scanning methods can achieve high spatial resolution through multiple measurements, albeit with much slower data acquisition and analysis. The recently proposed implicit speckle tracking methods provide a powerful tool to achieve fast and high throughput data processing thanks to the Fourier transform based analysis process. And the implicit speckle tracking methods have been improved to realize quantitative measurement under certain assumptions such as single-material and transparent material (Pavlov *et al.*, 2020*b* ▸), which is valid for biological samples under high X-ray energy. However, the implicit speckle tracking methods are not generally quantitative phase retrieval process, and carefully parameter selections are needed. Therefore, we restrict our research and comparison to within the general speckle tracking methods. High-efficiency X-ray imaging with high spatial resolution, especially for 3D imaging, remains imperative for exploring the intricate structures in bulk materials and their dynamic evolution. To address the need for speed, various techniques have been proposed to expedite data acquisition and analysis in scanning methods. Techniques such as wavelet-transform-based correlation and multi-resolution analysis have been proposed, offering over tenfold improvements in data analysis speed compared with conventional methods (Qiao *et al.*, 2020*b* ▸). And the well developed UMPA package with C language and parallel computation has shown significant improvement in data analysis speed, which accelerates the speckle tracking reconstruction process down to seconds (De Marco *et al.*, 2023 ▸). However, even with these advances, reconstruction time for full dataset in tomographic 3D imaging still spans tens of minutes, which falls short of the demands for high-speed 3D imaging feedback. The implicit speckle tracking methods might be a promising way to achieve real-time 3D reconstruction if the general quantitative measurement could be achieved in the future.

Advancements in deep learning (DL) have showcased remarkable potential in tackling complex calculations where analytical or numerical approaches often fall short. This transformative technology has significantly improved efficiency in various phase retrieval problems (Kemp, 2018 ▸; Benmore *et al.*, 2022 ▸; Rivenson *et al.*, 2019 ▸; Barbastathis *et al.*, 2019 ▸; Goy *et al.*, 2015 ▸). Previously, the SPINNet (Speckle-based Phase-contrast Imaging Neural Network) was developed as a deep learning model for single-shot SBI imaging, offering high accuracy and rapid data analysis. However, it suffers from reduced spatial resolution due to the single-image measurement approach (Qiao *et al.*, 2022 ▸; Shi *et al.*, 2023 ▸).

In this study, we introduce the Enhanced Scanning Pattern-based Imaging Neural Network (ESPINNet), designed to achieve a balanced performance between the spatial resolution and high speed in both data acquisition and analysis. ESPINNet has demonstrated exceptional performance across various scanning step sizes, trajectory configurations, and modulated pattern distributions, including speckle and periodic grating patterns, highlighting its adaptability to diverse experimental datasets. Furthermore, ESPINNet enables simultaneous dark-field image reconstruction, enhancing its capability for multi-contrast imaging. Moreover, ESPINNet is shown to be applicable to few number of scanning images required for high spatial resolution imaging, thus expediting the measurements and enabling high-speed 3D feedback in applications such as SBI and grating interferometry.

## 2. Methods

### 2.1. Physical model of the scanning pattern-based multi-contrast imaging

**Figure 1:**
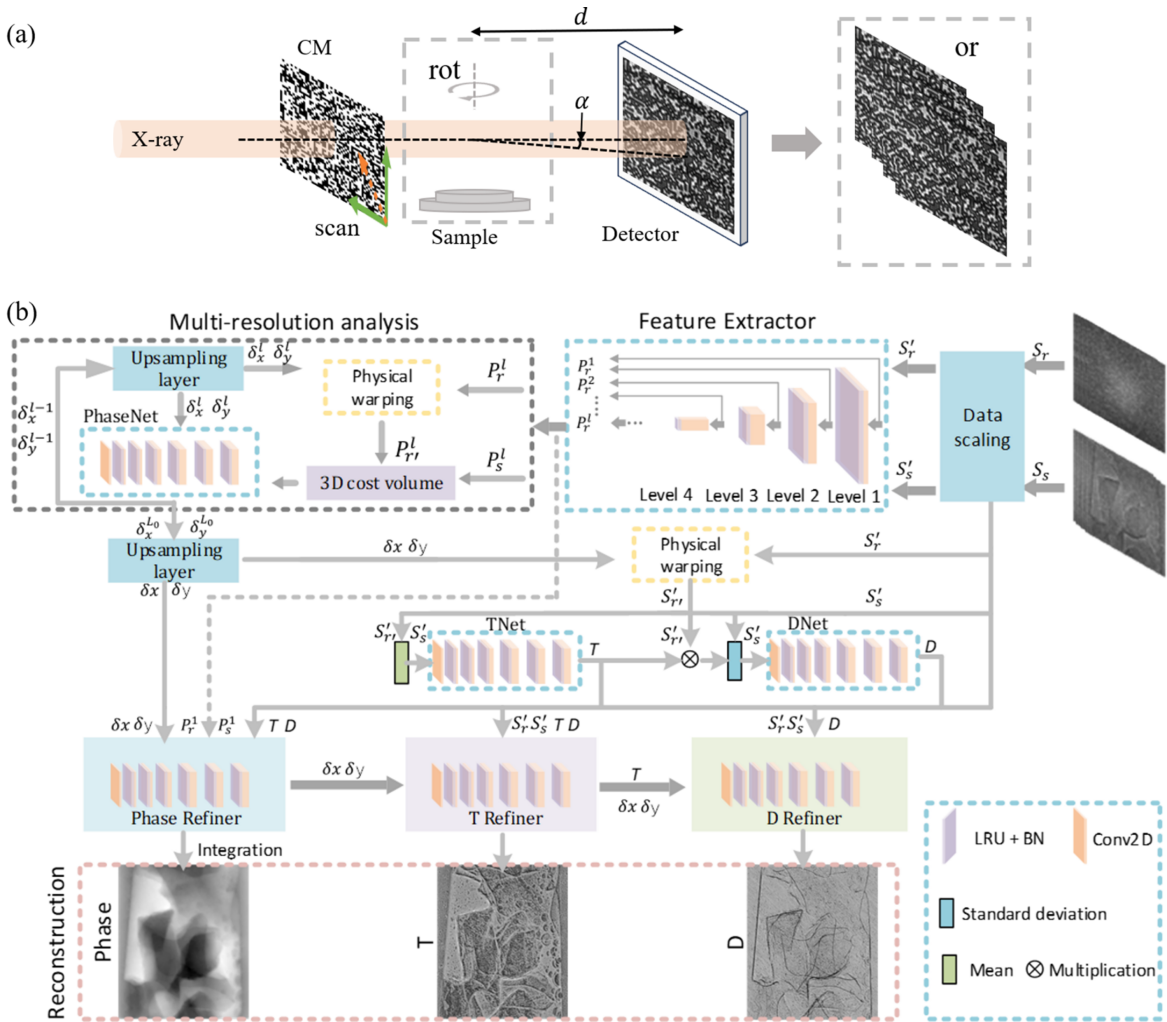
Schematic of the scanning pattern-based multi-contrast imaging. (*a*) Experimental setup. (CM: coded mask. The CM can be replaced with a Talbot grating or sandpaper as well. The CM can be scanned diagonally or in two dimensions.) (*b*) Network structure of the deep-learning model ESPINNet. (LRU: leaky ReLU layer; BN: batch normalization layer; Conv2D: 2D convolution layer.)

Fig. 1 ▸(*a*) illustrates the experimental setup for scanning pattern-based multi-contrast imaging, where *d* is the distance between the sample and the detector, and CM denotes the coded mask used to modulate the amplitude and phase of the incident X-ray beam, thereby generating the speckle distribution on the detector. While this discussion focuses on speckle-based multi-contrast imaging within the context of ESPINNet, it is important to highlight that the methodology can also be applied to other patterns generated by sandpaper, pinhole arrays, or gratings. Given the short wavelength and distance, the near-field propagation of the speckle or grating distribution can be simplified as geometrical propagation. Consequently, as the incident X-ray beam traverses the sample, it undergoes refraction at an angle α, which is directly linked to the sample’s phase distribution, expressed as (Berujon *et al.*, 2020*a* ▸; Zdora *et al.*, 2017 ▸) 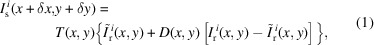
where 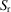 = 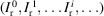 and 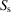 = 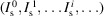 are the acquired pattern image stacks without and with the sample in the beam for scan *i* [*i* ∈ (1, *N*)], 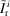 denotes the locally averaged intensity of the ‘reference’ image without sample, *T*(*x*, *y*) represents the transmission function of the sample, and *D*(*x*, *y*) is the small angle scattering of the sample, also known as ‘dark-field’, offering insight into the fine structure distribution beyond the spatial resolution. The displacements δ*x* and δ*y* in the pattern arise from the geometric propagation of X-rays induced by the sample phase ϕ, as illustrated below, 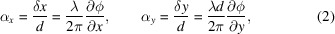
where α_*x*_ and α_*y*_ are the horizontal and vertical refraction angles induced by the sample’s phase. Once the speckle displacements δ*x* and δ*y* are recovered, the phase ϕ can be reconstructed using a 2D integration method such as Frankot–Chellappa, least-squares or Fourier-domain integration. Considering the time cost and accuracy, Frankot–Chellappa was implemented in the ESPINNet model, which could be found in the publicly available code (see the *Data availabilty* section at the end of the paper).

According to equation (1), scanning pattern-based imaging enables the recovery of phase ϕ, transmission *T*, and dark-field *D* by tracking the displacements of the speckle or grating pattern, intensity variation, and contrast variation in the local pattern intensity curves *I*_r_(*x*, *y*, *i*) or *I*_s_(*x*, *y*, *i*) (Berujon *et al.*, 2020*b* ▸; Wang *et al.*, 2016 ▸) for each scan *i*. For example, the speckle displacements δ*x* and δ*y* can be determined through the cross-correlation of the local speckle intensities in the ‘reference’ and ‘sample’ images, *I*_r_(*x*_0_, *y*_0_, *i*) and *I*_s_(*x*_0_ + δ*x*, *y*_0_ + δ*y*, *i*), respectively, for each pixel (*x*_0_, *y*_0_). The peak position of the cross-correlation gives the relative speckle displacements. Despite the power of modern workstations, the measurement and reconstruction of the speckle-tracking problem remain time-intensive due to two primary challenges: (1) inefficient cross-correlation computation, which demands significant time to track the speckle displacements for each pixel, especially in high-resolution images; and (2) a high dependence on the accuracy of the intensity line curve of the scanning speckle images for each pixel, necessitating a sufficient number of scan points *N* (typically on the order of tens), thus prolonging the SBI measurement and data analysis process.

### 2.2. Network structure of the ESPINNet

To address the challenges of data acquisition and analysis speed, we propose a deep learning model, ESPINNet, designed for rapid scanning pattern-based multi-contrast imaging. The architecture of ESPINNet is shown in Fig. 1 ▸(*b*), and consists of the ‘data scaling’, feature extractor, multi-resolution analysis, 3D cost volume and the refiner networks. To accommodate varying numbers of scans, the network integrates a ‘data scaling’ mechanism that adjusts the scanning pattern stacks *S*_r_ and *S*_s_ to a fixed number of scans *N*_net_, using linear interpolation, which are 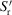 and 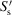. Based on experimental experience, *N*_net_ = 20 is selected to balance network size and data redundancy. For cases where the actual number of scans *N* < *N*_net_, linear interpolation is used, while for *N* ≥ *N*_net_, *S*_r_ and *S*_s_ are directly constructed using the first *N*_net_ raw images. In speckle-based imaging, the scan step is typically smaller than the speckle size, and reconstruction quality degrades as the step size is too large. Accordingly, selecting the first *N*_net_ images from each stack is preferable to uniform or random sampling, which would alter the effective step size for speckle tracking and potentially impair reconstruction.

The ‘feature extractor’ for capturing the high-dimensional structure of the speckle image stack comprises a multi-level convolutional neural network constructed with convolutional layers (Conv2D), leaky ReLU (LRU) nonlinear activation layers, and batch normalization (BN) layers, forming a standard encoder structure. The raw speckle image stack *S*_s_ with sample in the beam and the reference speckle image stack *S*_r_ without sample are pre-processed within the data-scaling part, then the ‘scaled’ image stacks 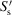 and 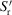 are the input of the feature extractor. Then the multi-level feature extractor could extract the coarse-to-fine structures 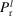 and 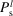 through the encoder structure, where *l* is the feature level. The extracted features 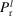 and 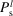 are subsequently used as input for the ‘multi-resolution analysis’ at level *l*, integrating physical warping and the 3D cost volume to establish a correlation between the speckle or grating displacements (δ*x* and δ*y*) and the extracted features. The physical warping process is a simplified version of equation (1) with *D*, and can be expressed as 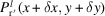 = 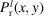. Therefore, the features with sample 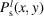 corresponds to the pixel-shifted reference features 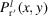.

The use of the 3D cost volume allows local cross-correlation for the prediction of speckle displacements. By applying physical constraints such as physical warping and 3D cost volume, derived from the correlation-based methods for speckle tracking analysis, ESPINNet achieves enhanced generalization and improved accuracy.

The ‘upsampling layer’ is specifically designed to scale the images or speckle displacements obtained from the higher level (*l* + 1) to the appropriate resolution. Subsequently, PhaseNet utilizes the extracted features and the 3D cost volume, which contains local correlation information of the speckle distribution, to predict the speckle displacements. Following the ‘multi-resolution analysis’, the speckle displacements can be accurately retrieved, and the ‘upsampling layer’ is utilized to resize the speckle displacements from the lower levels to the highest resolution. For the lowest level of the multi-resolution analysis, the speckle displacements are generated as zero for the physical warping process. Additionally, DNet and TNet are compact convolutional neural networks designed to predict the dark-field and transmission distributions based on the predicted speckle displacements and the scanning pattern images. It should be noted that the DNet and TNet do not implement the multi-resolution analysis, and the transmission and scattering image are predicted using the DNet and TNet based on the speckle displacements δ*x* and δ*y* from the PhaseNet and the speckle patterns 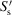 and 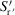 which is obtained from the physical warping process, and the mean and standard variation of 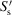 and 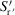 are used for the TNet and DNet, respectively.

In the final stage, the refiner sub-networks play a crucial role in refining the speckle displacements, transmission, and dark-field images by eliminating noise and artifacts. The ‘upsampling layer’ consists of nearest-neighbor interpolation combined with a Conv2D layer with a 3 × 3 kernel size, instead of simpler interpolation methods. This design choice was made to enhance the achievable spatial resolution (Seichter *et al.*, 2021 ▸). Compared with a basic bilinear interpolation method, the network employing the ‘upsampling layer’ demonstrated better performance. By integrating a 3 × 3 depth-wise convolution, the network effectively integrates neighboring features, thereby improving the reconstruction quality. This improvement can be attributed to the learnable nature of the convolution layer, which adapts to the specific requirements of the speckle tracking task during network training.

The proposed ESPINNet is trained using the following loss function,
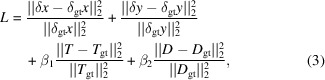
where δ_gt_*x* and δ_gt_*y* are the ground truth speckle displacements, and *T*_gt_ and *D*_gt_ represent the ground truth transmission and dark-field scattering, respectively. The coefficients β_1_ and β_2_ are used to balance the loss function of *T* and *D* to the speckle displacements (δ*x* and δ*y*). During training, the values of β_1_ and β_2_ are set to 10 to optimize the training process.

### 2.3. Detailed network structure of ESPINNet

The ESPINNet consists of feature extractors to extract high-dimensional structures from the scanning images, the estimators (PhaseNet, TNet, and DNet) to retrieve the multi-contrast information, the refiners (PhaseRefiner, TRefiner, and DRefiner) to remove noise and artifacts, the warping layer connecting the reference and sample images based on a physical model, the 3D cost volume layer to provide local correlation constraints for the speckle displacement prediction, and the learnable upsampling layer to scale the lower-level multi-resolution analysis to high-level analysis. The multi-resolution analysis has been widely used in various neural networks for computer-vision tasks (Seichter *et al.*, 2021 ▸) and demonstrates good performance with coarse-to-fine analysis. In the ESPINNet, the multi-resolution analysis can significantly reduce the computational cost and improve the inference accuracy by increasing the effective searching window size, which has been used in speckle tracking analysis as well (Qiao *et al.*, 2020*a* ▸).

**Figure 2:**
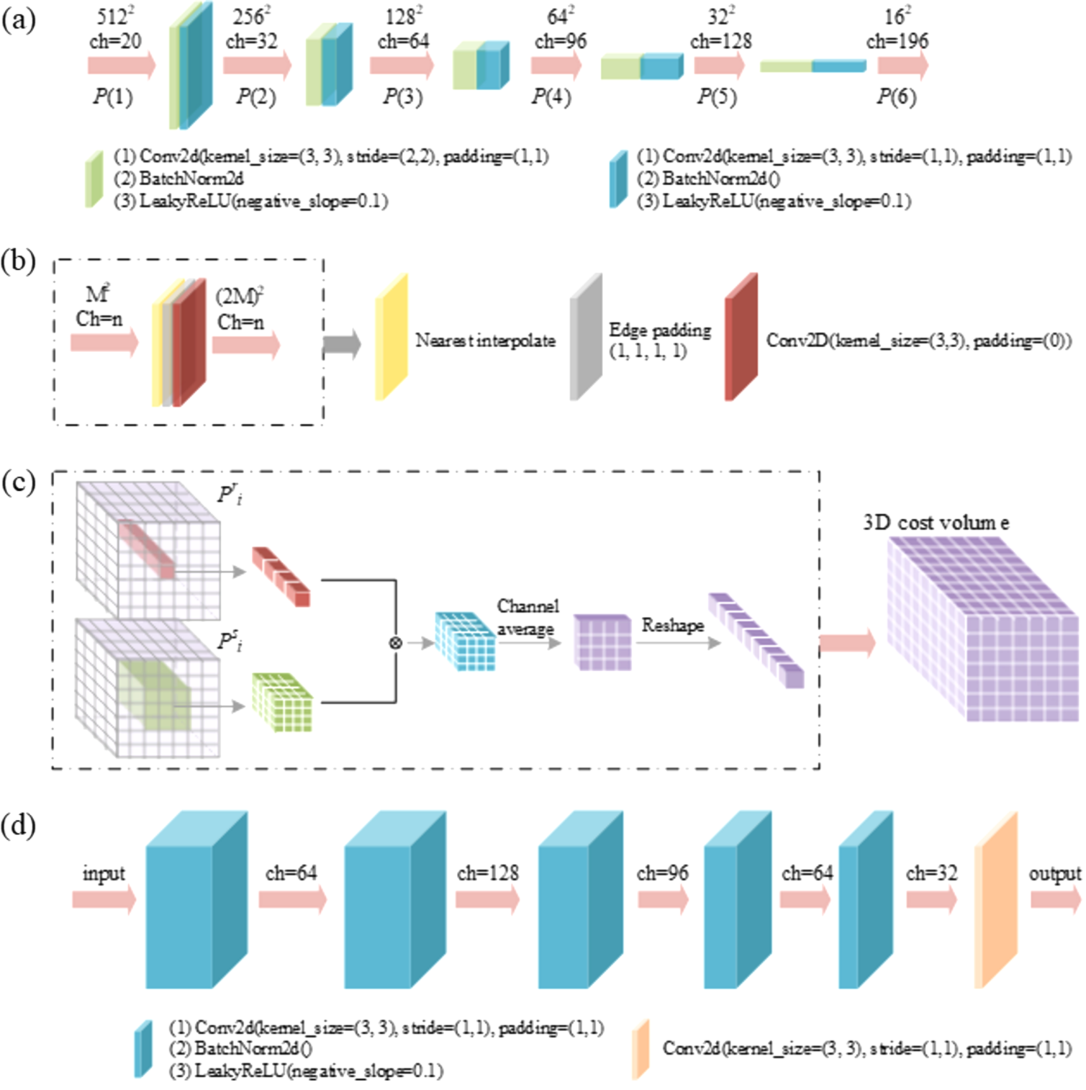
Detailed sub-network structures of ESPINNet. (*a*) Feature extractor; (*b*) learnable upsampling layer; (*c*) 3D cost volume generation; (*d*) sub-network structure of estimators (PhaseNet, TNet, DNet) and refiners (PhaseRefiner, TRefiner, DRefiner).

Fig. 2 ▸ shows the detailed structures of the sub-networks used in ESPINNet. The convolutional neural network (CNN) with a typical encoder structure is used in the feature extractors, shown as the purple block in Fig. 2 ▸(*a*), and consists of a 2D convolution layer (Conv2d) with a stride of 2, a 2D batch normalization layer (Batchnorm2d), and a leaky Relu nonlinear activation layer (LeakyRelu). The blue block has a similar structure but a Conv2d layer with a stride of 1. The whole block down-samples the input data and maps the spatial information into the channel dimension. For example, the input image stack has a spatial size of 512 × 512 with 20 channels (the channel number corresponds to the scanning points during measurement) and is down-sampled by a factor of 2 with an increasing channel number from 20 to 32, which will be used for the multi-contrast estimators. During the network training, a multi-resolution level of 5 was used, so that the training data are encoded into a 3D volume with a size of 16 × 16 × 196. The reference and sample image stacks share the same feature extractor networks to reduce network size.

**Figure 3:**
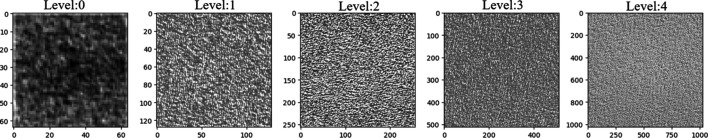
Example of the extracted feature images for different pyramid levels.

Fig. 3 ▸ shows an example of the extracted feature images from the speckle image stacks for a single channel. The input speckle images have dimensions of 2048 × 2048 pixels, and the highest level 1could down-sample the speckle images and extract fine speckle features as shown in the figure for level 2. For the lower pyramid level, the sizes of the feature images reduce gradually and coarser structures are extracted.

The structure of the upsampling layer is shown in Fig. 2 ▸(*b*), consisting of the nearest interpolation operation and the edge padding to remove the edge artifacts and a Conv2D layer with a kernel size of 3, where *M* is the image size. Compared with the simple linear or bilinear interpolation, the learnable upsampling layer can retain more details and allows less feature degradation during the image scaling process and has been used in areas such as super-resolution, segmentation, and object tracking. During the training of the ESPINNet, the upsampling layer not only grasps the feature of the speckle images but also adapts to diminish artifacts while preserving high-frequency structures.

The 3D cost volume was designed to correlate the feature distribution of two images or stacks, enabling object tracking and recognition in computer vision. Remarkably, this process is similar to the local speckle displacement search employed in speckle-tracking methods through cross-correlation. Therefore, the 3D cost volume is implemented in the ESPINNet to add the physical constraints for the PhaseNet to recover the speckle displacement or refraction. The detailed structure of 3D cost volume is shown in Fig. 2 ▸(*c*). The reference and sample feature volume generated by the feature extractors are correlated ‘pixel-wise’ within the ‘height’ and ‘width’ dimensions and along the channel dimension. For example, the single ‘pixel’ of the reference volume 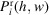 is multiplied by every ‘pixel’ of the sample volume 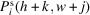 to get the correlated volume, where *k*, *j* ∈ (−*n*_w_, *n*_w_) and *n*_w_ is the ‘searching window’ in the speckle tracking methods. Combined with the multi-resolution analysis, *n*_w_ = 3 is sufficient to accurately reconstruct speckle movement. Then the correlated volume is averaged along the channel dimension and reshaped into a 1D line, which is a simplified correlation when the distribution is zero-biased. The above process is repeated for every pixel in the feature volumes and the obtained 1D lines for every pixel are rearranged into the final 3D cost volume.

The estimators and refiners, shown in Fig. 2 ▸(*d*), play a pivotal role in reconstructing multi-contrast images by leveraging the encoded structure information obtained from the feature extractors. This process is designed based on the decoder structure of the CNN architecture. The single block (blue block) consists of a Conv2D layer with a stride of 1 and a kernel size of 3, a batch normalization layer, and a leaky ReLu nonlinear activation layer, and the final output block (yellow block) is a single Conv2D layer with a kernel size of 1 to avoid the spatial resolution degradation caused by the convolutional operation. In the ESPINNet, each estimator and refiner uses the same structure as mentioned above, with the output channel set at 1 for transmission *T* and scattering *D*, and 2 for ‘phase’ δ_*x*_/δ_*y*_.

### 2.4. Simulated training data generation process

In deep learning models, the training data quality significantly impacts the network’s performance and generalization ability. For ESPINNet, a simulation dataset of 10000 samples with diverse phase and absorption distributions is generated, following the physical model described in equation (1). After training, a small set of experimental data is used as a test dataset to assess the network’s performance on real-world data.

The procedure of the simulated training data generation is as follows: the random mask pattern is first generated with a pitch size of 5 µm; then the reference image is generated according to the near-field Fresnel propagation; the sample image generation uses the reference image based on equation (1), while the sample phase, absorption, and scattering information are added before the propagation to the detector plane. The samples are generated randomly through the following steps: (1) use the Bézier curve to generate the outline of the sample with random structures; (2) generate random phase, transmission and scattering from random pixel-wise noise and use the Gaussian filter to smooth the noise distribution so that the highest spatial frequency of the sample distribution is below the resolution at detector plane, which is 1 µm here; (3) fill the sample outline with the generated phase, transmission and scattering distribution. It should be noted that the peak variation of the sample’s phase, transmission, and scattering randomly fluctuates within a desired range determined by the experimental conditions, so that the sample–detector distance can be a fixed value to simplify the dataset. The simulated training data were generated from equation (1) with the following parameter ranges: 0.02 ≤ *T* ≤ 0.2; 0.02 ≤ *D* ≤ 0.2; 0 ≤ ϕ ≤ 10π; and feature sizes in the range of [0, 10π] pixels.

Based on the experimental experience, a scanning number of *N*_net_ = 20, a scanning step size of 3 µm, and a virtual pixel size of 0.65 µm are selected. For the scanning pattern, the horizontal (*X*) and vertical (*Y*) positions were constructed using a fixed step size with an added random offset of ±0.5 step size. All random variables were drawn from uniform distributions. In this case, the scanning step size is not an integer multiple of the pixel size to make the training dataset more representative of real-world application scenarios. The sub-pixel speckle movement is calculated using the Fourier-based image-shifting method. It is noteworthy that, for the sake of simplicity, the simulation data generation process utilizes a diagonal linear scan pattern. Nonetheless, the trained model is versatile and can be directly applied to 2D scanning patterns or nonlinear scanning patterns. A total of 10000 samples were generated in the simulation dataset, with 80% of these samples utilized as the training dataset, while the remaining samples were allocated for the validation dataset.

### 2.5. Network training procedure

The network underwent training in multiple stages, starting from the low multi-resolution level 4 and progressing to the highest level, excluding the refiner sub-networks. The training epochs for each stage were set at 200, 500, and 800, respectively. Subsequently, the multi-resolution level 1, in conjunction with the refiner sub-networks, was trained for a total of 1500 epochs. The Adam optimizer with a learning rate of 10^−4^ was used for network training optimization. Due to the large size of the training images (512 × 512 pixels), the network itself is considerably large, necessitating the use of 8A100 GPUs for the training process for 27 h. The batch sizes for each level are 128, 72, 64, and 64, which are determined based on the available GPU memory.

### 2.6. Experimental setup

After training on the simulation dataset, ESPINNet was validated using experimental data acquired at two synchrotron facilities: the 1-BM beamline of the Advanced Photon Source (APS), Argonne National Laboratory, USA, and the BL13HB beamline of the Shanghai Synchrotron Radiation Facility (SSRF), China. All the experimental setup parameters are shown in Table 1 ▸.

At the 1-BM beamline of APS, the X-ray energy was set to 14 keV using a Si(111) double-crystal monochromator. The sample-to-detector distance was 0.5 m, and the speckle patterns were generated using a coded mask with a pitch size of 5 µm. The coded mask was patterned using electron beam lithography on a silicon nitride membrane with a thickness of 2 µm, significantly improving speckle contrast compared with conventional speckle generators such as sandpaper and membranes, thus enhancing its suitability for SBI applications (Qiao *et al.*, 2021 ▸). The detector setup consisted of a 10× objective, a LuAG:Ce scintillator (50 µm thickness), and an Andor Neo sCMOS camera.

At the BL13HB beamline of SSRF, the X-ray energy was set to 20 keV using a double multi-layer monochromator (DMM). The sample-to-detector distance was 0.5 m, and the grating patterns were generated using a Talbot checkerboard grating with a period of 20 µm. The detector setup included a 10× objective, a LuAG:Ce scintillator (100 µm thickness), and a Flash 4.0 sCMOS camera (Hamamatsu).

Simulation data for ESPINNet training were generated based on the coded mask pattern. Notably, the network demonstrated robust performance when tested with data generated by both the coded mask and Talbot grating, as well as with a conventional sandpaper speckle generator, across varying X-ray energies and sample-to-detector distances, as detailed below.

## 3. Experimental results

### 3.1. Quantitative characterization of ESPINNet

One advantage of the modulated-pattern analysis method is its ability to provide quantitative measurements, which distinguishes it from other methods like the propagation-based approach. In this study, the phase reconstruction accuracy of ESPINNet was evaluated against that of the conventional speckle tracking method. The test sample was a standard beryllium lens with an apex radius of 200 µm. The coded mask was scanned using a 10×10 2D grid pattern, with Gaussian random step size, characterized by a mean of 3 µm and a standard deviation of 0.3 µm. Initially, the phase was reconstructed using the X-ray speckle vector tracking method (XSVT) with all 100 scanning positions, serving as ground truth for precise measurements. Subsequently, 20 scanning images were sequentially chosen from the original set of 100 images for both ESPINNet and XSVT methods.

**Figure 4:**
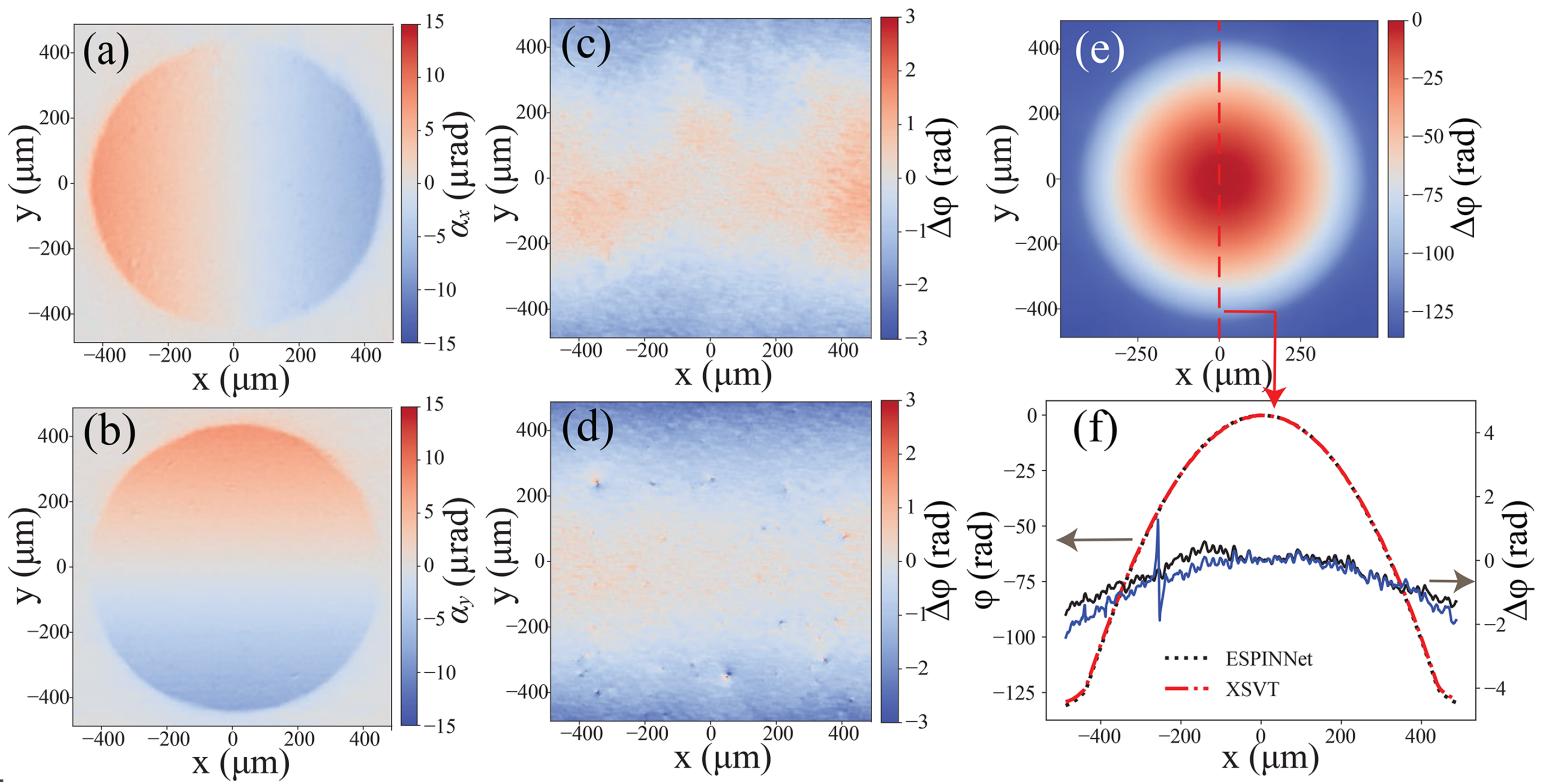
Phase reconstruction of a beryllium CRL (*R* = 200 µm) at 14 keV with 5 µm coded mask using ESPINNet and XSVT. (*a*, *b*) Predicted horizontal and vertical refraction using ESPINNet. Panels (*c*) and (*d*) show relative phase errors of ESPINNet and XSVT with *N* = 20 compared with the ground truth (XSVT with *N* = 100), respectively. (*e*) Predicted phase using ESPINNet. (*f*) Vertical line profile of the phase (red dashed line: phase profile of the ground truth; black dashed line: phase profile retrieved using ESPINNet; black solid line: relative phase error of ESPINNet compared with ground truth; blue solid line: relative phase error of XSVT with *N* = 20 compared with ground truth). The 5 µm coded mask was scanned with a step size of 3 µm and an ‘*XY*’ 2D pattern. Data were acquired at the 1BM beamline of APS.

The reconstruction of the beryllium lens is shown in Fig. 4 ▸, where (*a*) and (*b*) are the predicted horizontal and vertical refraction of the ESPINNet reconstruction, respectively. In Figs. 4 ▸(*c*) and 4(*d*), the relative phase errors of ESPINNet, the XSVT method with *N* = 20 are compared against the ground truth (XSVT with *N* = 100). Notably, the XSVT with *N* = 20 shows more noise and spikes than the ESPINNet reconstruction. In Fig. 4 ▸(*f*), the line profile of relative phase errors of the reconstructed phases using ESPINNet and XSVT with *N* = 20 are represented by the black and blue solid lines, respectively. The ESPINNet line profile exhibits an RMS error of 1/11λ, while the XSVT method with *N* = 20 achieves 1/9λ, suggesting that the prediction accuracy of the ESPINNet model is comparable with or even exceeds that of the XSVT method. Furthermore, the predicted phase from the ESPINNet demonstrates significantly less noise compared with the XSVT method, emphasizing the enhanced noise reduction capability of the ESPINNet.

### 3.2. Comparison of data scaling methods for ESPINNet

**Figure 5:**
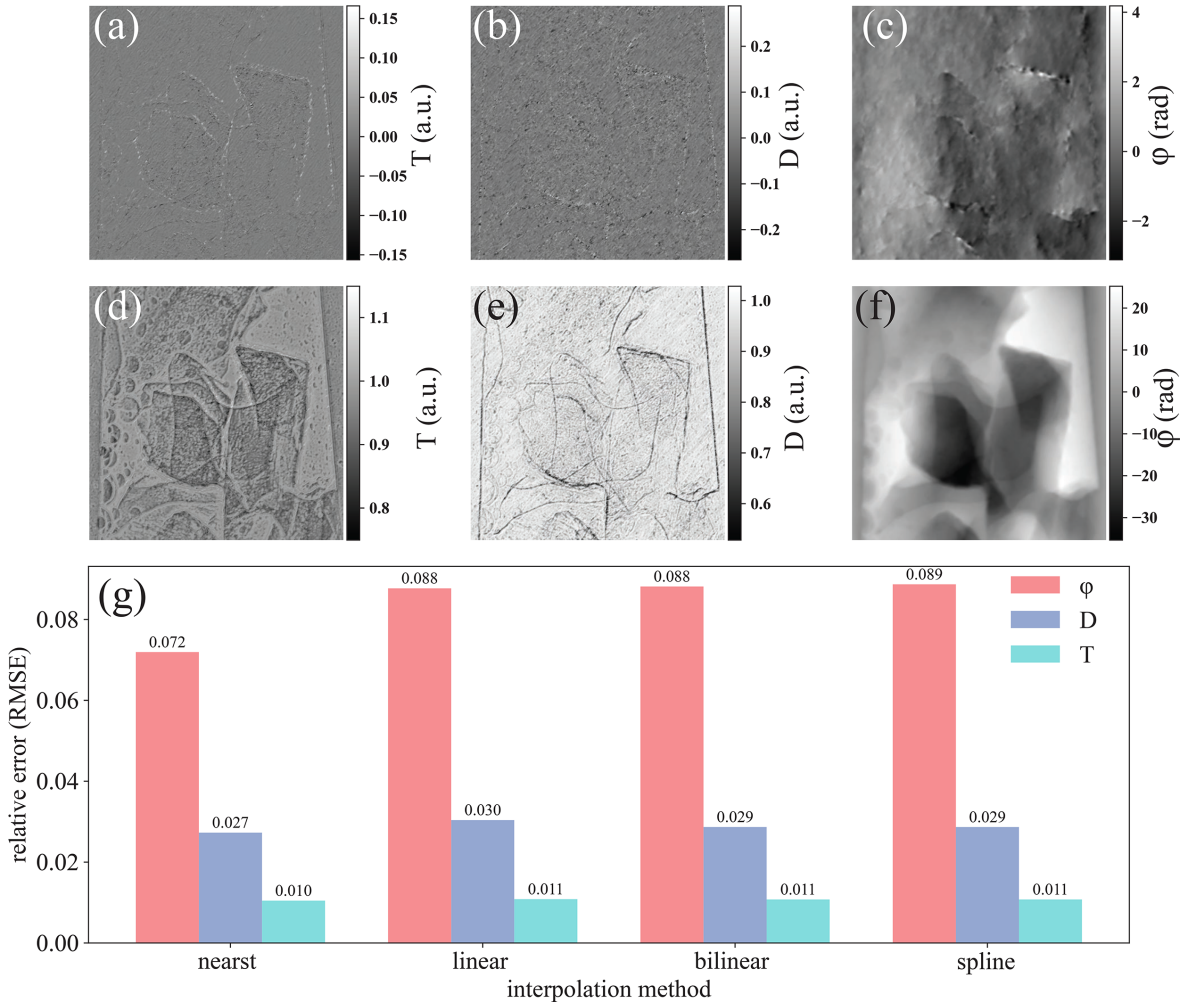
Results for coal using different data scaling methods. (*a*–*c*) Recovered error of *T*, *D*, and phase with *N* = 10 using nearest interpolation; (*d*–*f*) recovered *T*, *D*, and phase with *N* = 20; (*g*) relative mean square error with nearest, linear, bilinear and spline interpolation compared with the ‘ground truth’ using *N* = 20.

ESPINNet was designed and trained with *N* = 20 projections. Consequently, input stacks are either truncated to the first 20 images when *N* > 20 or upsampled to 20 when *N* < 20. Because data scaling can influence reconstruction quality, we evaluated several interpolation strategies using a coal–water sample (Fig. 5 ▸). Specifically, data acquired with *N* = 10 were resampled to *N* = 20 using nearest, linear, bilinear, and spline interpolation, and we computed the relative mean square error for transmission, dark-field, and phase (Fig. 5 ▸).

Among the tested methods, nearest interpolation produced the lowest error; the other methods yielded slightly higher errors. The spatial distributions of the relative errors for transmission, dark-field, and phase shown in Figs. 5 ▸(*a*)–5(*c*) (computed between *N* = 10 with nearest scaling and the native *N* = 20 data) indicate that discrepancies are concentrated near sample edges, where transmission and phase vary rapidly. Nevertheless, the quantitative differences among the four interpolation methods are small [Fig. 5 ▸(*g*)]. We attribute this insensitivity to the network’s feature extraction, which appears to suppress noise and interpolation-induced artifacts introduced during data scaling.

### 3.3. Reconstruction performance and speed

The data analysis speed and experimental efficiency stand as a critical challenge in achieving real-time imaging. The measurement speed is determined by factors such as the exposure time per image, the stage scanning velocity, and the total number of scanning positions. The exposure time, dictated by X-ray flux, can be less than 1 s with an undulator beamline. Nonetheless, scanning speckle tracking measurements demand tens of scanning positions (*N*) if high resolution is demanded, extending the measurement time to minutes. By implementing the proposed ESPINNet approach, the number of scanning positions (*N*) can be significantly reduced without sacrificing measurement precision and quality, thereby streamlining the experimental process.

**Figure 6:**
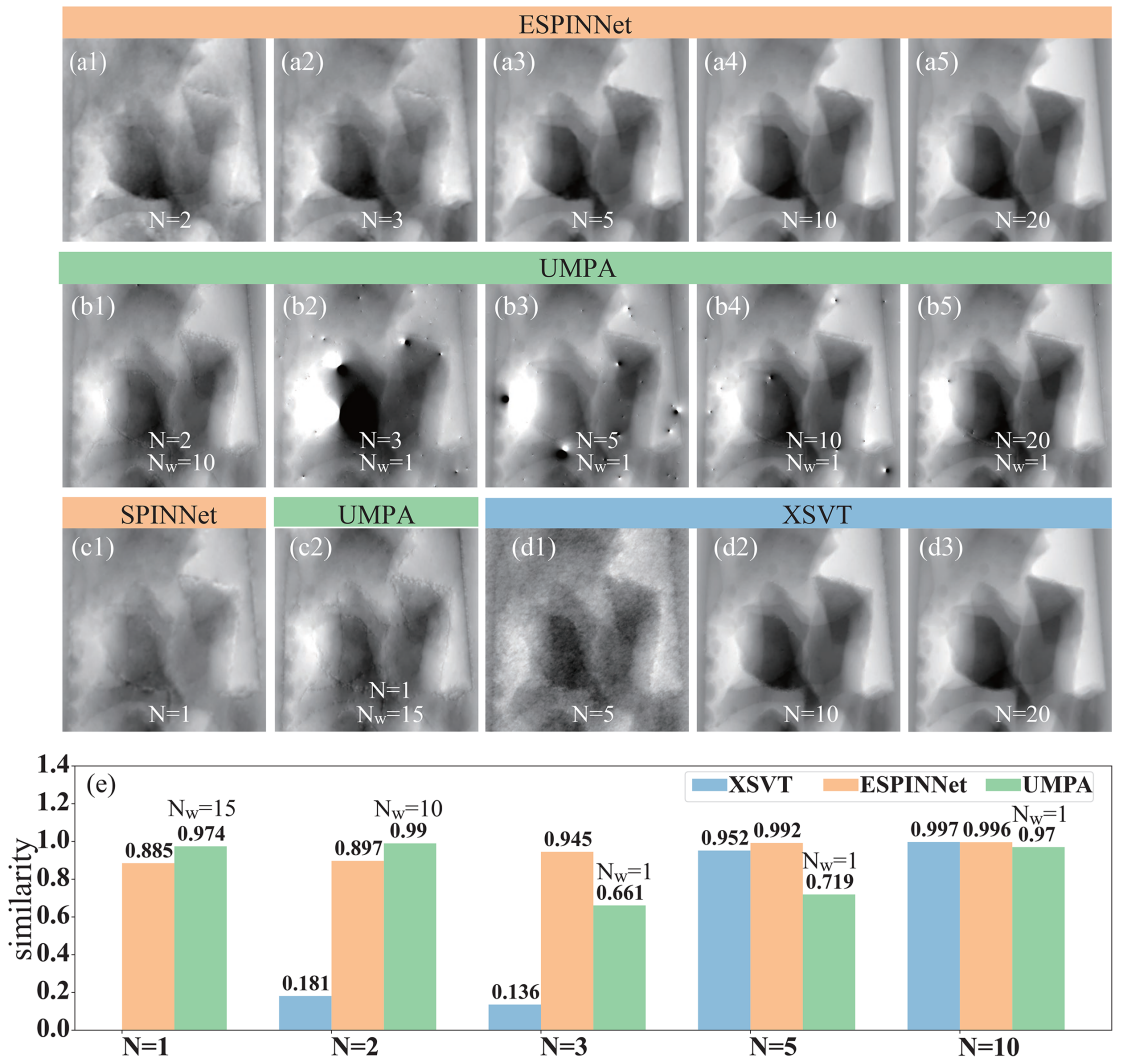
Reconstruction comparison with varying scan number *N*. (*a*1–*a*5) Recovered phase using the ESPINNet with *N* = 2, 3, 5, 10, 20; (*b*1–*b*5) Recovered phase using the UMPA with *N* = 2, 3, 5, 10, 20, while the window size used in UMPA is 10 for *N* = 2 and 1 for others; (*c*1) recovered phase using SPINNet with *N* = 1; (*c*2) recovered phase using UMPA with *N* = 1 and *N*_w_ = 15 [*N*_w_ is the analysis window size as described by De Marco *et al.* (2023 ▸)]; (*d*1–*d*3) recovered phase using the XSVT method with *N* = 5, 10, 20; (*e*) similarity of the recovered phase using different scan number *N* compared with scan number *N* = 20 for XSVT, UMPA, and ESPINNet. Data were acquired at the 1BM beamline of APS.

Fig. 6 ▸ shows the phase reconstruction using XSVT, UMPA and ESPINNet across varying numbers of scanning positions *N*. In this figure, panels (*a*1)–(*a*5) are the phase images reconstructed using ESPINNet, (*b*1)–(*b*5) are the phase images obtained from UMPA with window size of 10 for *N* = 2 and 1 for others, (*c*1) is the phase obtained using SPINNet with *N* = 1, while (*c*2) is the phase image using UMPA with *N* = 1 and *N*_w_ = 15. Panels (*d*1)–(*d*3) display phase images reconstructed using the XSVT method, and (*e*) shows the similarity (cross-correlation) of phase results obtained with different scan numbers compared with that obtained using *N* = 20. For the UMPA method, a window size of 1 was used for cases with *N* ≥ 3 to achieve the best spatial resolution. However, for *N* = 1 and *N* = 2, the window size must be increased to 15 and 10, respectively; otherwise, the reconstruction fails. It should be noted that the network structure of ESPINNet is designed for scanning mode with *N* ≥ 2, while the SPINNet network is applicable for ‘single mode’. Therefore, we treat the SPINNet as the special case of ESPINNet for *N* = 1 here. The results highlight the substantial superiority of ESPINNet’s phase reconstruction in Figs. 6 ▸(*a*3)–6(*a*5) over the XSVT method in (*d*1)–(*d*3), especially evident at fewer scanning positions. As the scanning number decreases, the XSVT method starts to fail when *N* is less than 10, whereas the ESPINNet model can still provide acceptable phase results with low noise. Since the XSVT method is based on the vector tracking process, it is reasonable that the reconstruction performance degrades along with fewer scanning points. Furthermore, phase similarity metrics affirm the precise prediction of the ESPINNet at *N* = 3. From the insights garnered in Fig. 6 ▸, it is evident that ESPINNet is adept at processing speckle images acquired with three times fewer scanning positions, resulting in a measurement speed improvement of about two to three times compared with the XSVT method. However, it should be noted that the XSVT method is a special case for *N*_w_ = 0 where a single pixel is calculated instead of a window. By using a window size *N*_w_ > 0, the speckle tracking methods can accommodate fewer scanning positions theoretically. Here we compare the results with the UMPA method as shown in Figs. 6 ▸(*b*1)–6(*b*5). As the scanning number *N* decreases, more artifacts appear in the phase image using *N*_w_ = 1 (a typical parameter setting for UMPA) for the best resolution. As expected, the UMPA with *N*_w_ = 1 starts to fail when *N* ≤ 5 as shown in Fig. 6 ▸(*e*). By increasing the window size *N*_w_, the UMPA method works well for *N* = 2 and even *N* = 1 with nearly no artifacts as shown in (*b*1) and (*c*2). However, the spatial resolution of UMPA with large window size, *N*_w_ = 10 and 15 for *N* = 2 and 1, is lower than the phase image with *N*_w_ = 1 in (*b*5) and ESPINNet in (*a*1) and (*a*2) due to the low-pass filtering effect. Considering that the multi-resolution analysis is included in the ESPINNet structure, it is not suprising that the ESPINNet can work with fewer scanning positions. According to the reconstructed results in Fig. 6 ▸, the ESPINNet provides a balanced performance between the experimental speed with fewer scanning positions *N* and image quality, for example spatial resolution and fewer artifacts, compared with the XSVT and UMPA methods. It is interesting to see that the phase image using ESPINNet with *N* = 2 shows more details than SPINNet with *N* = 1 in Fig. 6 ▸(*b*), especially the area near the edge where the phase jump exists, while the phase from SPINNet has less noise due to the inherent ‘smoothing effect’ within the network structure. In a word, the SPINNet for a single image and ESPINNet for scanning mode provide a powerful tool for modulated pattern analysis, which outperforms the correlation-based calculation methods and gives a balanced performance in speed, quality, and implementing difficulty.

The speed of the multi-contrast imaging is predominantly constrained by the data analysis required to extract multi-contrast images from the scanning speckle data and the pace of experimental data collection, which is strongly dependent on the scan number *N* performed. Firstly, the data analysis speed of ESPINNet was evaluated against the XSVT method and the UMPA method. Efforts to accelerate the XSVT data processing involved leveraging CPU-based parallel computing and optimizing core functions using a JIT (just-in-time) compiler. The UMPA is implemented using the highly optimized open-source code of De Marco *et al.* (2023 ▸). The comparison setup featured a workstation equipped with two dual-socket Xeon 8462Y+ Platinum processors, 512 GB RAM, and an RTX A6000 GPU tasked with handling the DL model prediction. For a dataset consisting of *N* = 20 speckle images, each with an array of 2048 × 2048 pixels, the XSVT method took 95 s to process, while the UMPA with *N*_w_ = 1 and 128 threads in dark-field mode took 0.997 s and ESPINNet deployed using TensorRT accomplished the task in only 0.2 s with a batch size of 1 marking a good speed enhancement. Detailed benchmarking results are provided in Table 2 ▸. After TensorRT optimization with float 16, the model size is only 14 MB, and the trained ESPINNet contains 4.13 million parameters. As shown in Table 2 ▸, GPU memory usage during inference increases with both batch size and image resolution: for example, a 2048 × 2048 input requires 2.4 GB for a batch size of 1, but 31.2 GB for a batch size of 16. Inference throughput benefits from larger batch sizes. For instance, at 2048 × 2048, the per-image-pair latency is 85 ms with a batch size of 10. For smaller inputs such as 512 × 512, a batch size of 32 requires 4 GB of GPU memory and yields a per-image-pair time of 8.6 ms. These results indicate that ESPINNet is well suited for real-time analysis. Although the RTX A6000 GPU used is considered relatively outdated compared with the current top-tier GPU computing cards, its performance sufficed for fast data analysis. It is important to note that these execution times reflect the optimal deployment environment for each specific algorithm rather than a unified hardware benchmark. UMPA is currently implemented only for CPU architectures and was therefore tested on a high-performance 128-thread node to maximize its potential, which could be possibly improved using GPU hardware. In contrast, ESPINNet is designed for GPU acceleration; running it on a CPU would not reflect realistic usage patterns. Thus, the comparison highlights the practical time-to-solution for users operating each tool in its intended environment. The prospect of achieving real-time data analysis appears promising by integrating the ESPINNet into edge computing hardware with a comparable computing performance, and a light-weight version of ESPINNet optimized for inference speed could be developed for real-time imaging applications.

### 3.4. Generalization capability of ESPINNet

**Figure 7:**
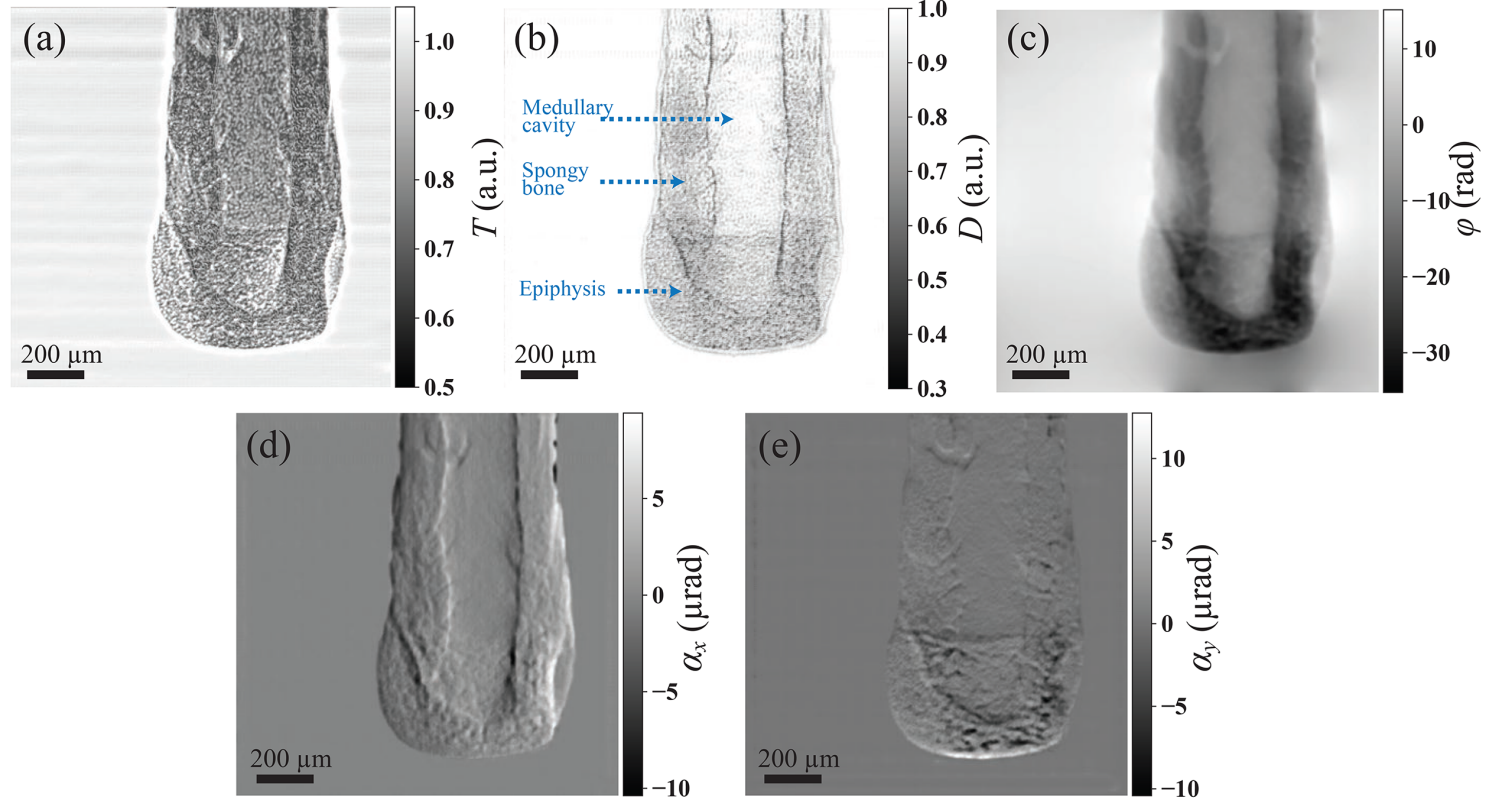
Reconstructed multi-contrast information of a mouse bone using ESPINNet from single-grating interferometry data acquired with a step size of 6 µm and a linear diagonal scanning pattern. (*a*) *T*: transmission; (*b*) *D*: scattering; (*c*) ϕ: phase; (*d*) horizontal refraction; (*e*) vertical refraction. Data were acquired at the BL13HB beamline of SSRF.

While DL-based methods have found widespread application in diverse imaging techniques, concerns persist regarding generalization capability, whether a model learns the physical principle or merely fits the training data. To validate the generalization ability of the ESPINNet, the ESPINNet model trained on speckle data was directly used to reconstruct the multi-contrast images from a single-grating interferometer. This dataset featured a checkerboard grating with a pitch size of 20 µm, scanned in the 2D grid ‘*XY*’ direction with a step size of 6 µm at an X-ray energy of 20 keV. Reconstructed images of a mouse bone, shown in Fig. 7 ▸, exhibit high-quality simultaneous retrieval of transmission, scattering, and phase. The precision of the refraction angle, quantified as the RMS of the blank region in the reconstructed refraction image, is 85 nrad. Notably, the phase image demonstrates superior contrast, while the scattering image delineates density disparities of the inner medullary cavity and outer structure of spongy bone and epiphysis, showcasing the benefits of multi-contrast imaging. The successful application of a model trained on simulated speckle data to experimental grating-based data suggests that the network has learned the fundamental physical principles of wavefront modulation, rather than overfitting to specific training patterns. Despite being trained on a simulation dataset comprising scanning speckle images, the ESPINNet exhibits promising adaptability to experimental images, including both scanning speckle images and grating images. While a systematic quantification of generalization across all possible experimental conditions remains to be established, these results provide a compelling proof-of-concept for cross-modality application. In addition, we evaluated ESPINNet on three datasets—X-ray compound refractive lenses (CRLs), a coal–water sample, and mouse bone—acquired using an *XY* 2D grid, a linear diagonal pattern, and an *XY* 2D grid, respectively. These results further indicate that ESPINNet is largely insensitive to the speckle-image scanning pattern and exhibits strong generalization capability. Collectively, these findings imply that, once trained, the model holds potential for real-world applications in modulated pattern analysis without necessitating transfer learning or continuous learning.

### 3.5. Multi-contrast 3D imaging using ESPINNet

**Figure 8:**
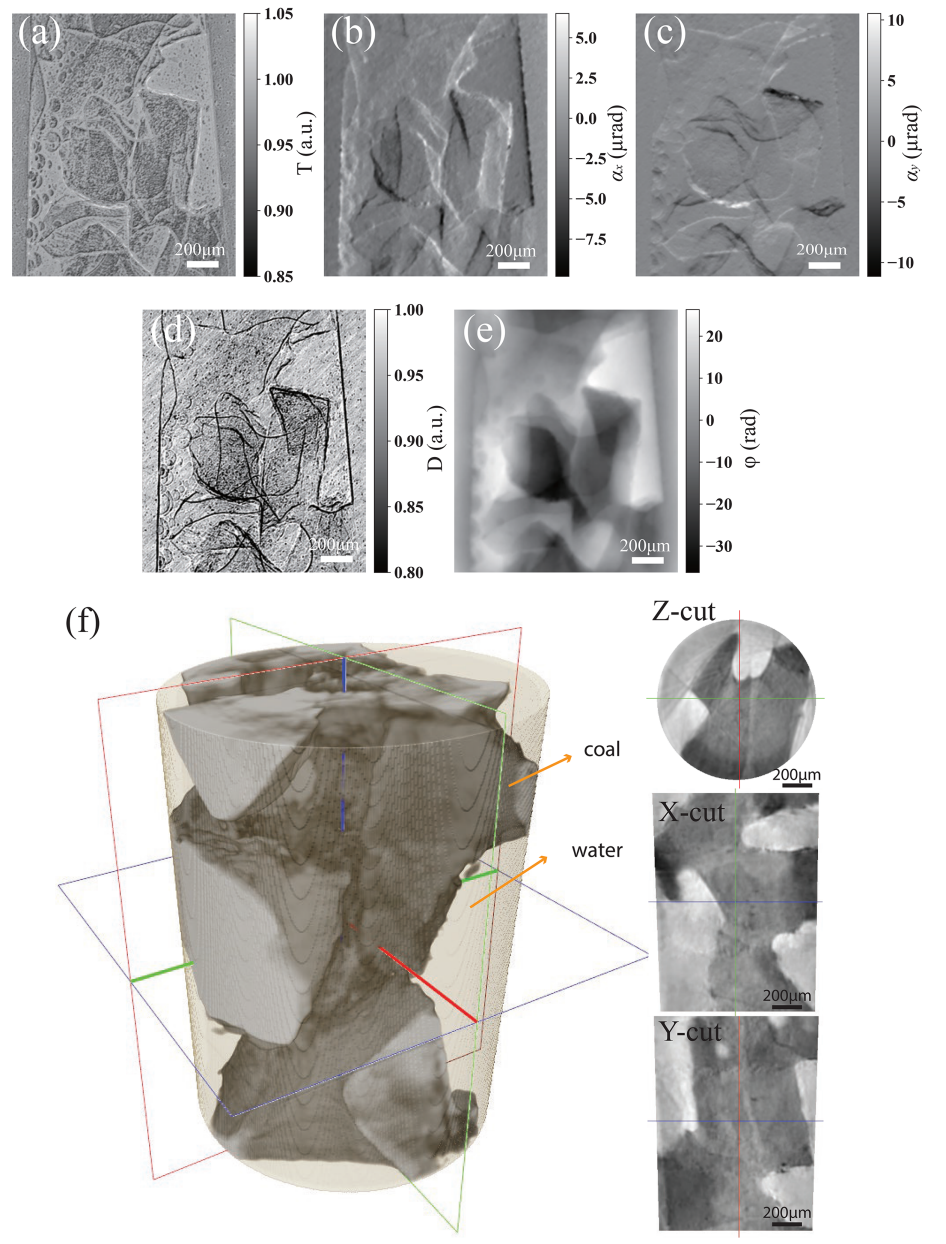
Reconstruction of a coal and water mixed sample. (*a*–*e*) Multi-contrast images using ESPINNet with *N* = 20 of *T* (transmission), α_*x*_ (horizontal refraction), α_*y*_ (vertical refraction), *D* (scattering), and phase distributions; (*f*) 3D phase volume using ESPINNet with *N* = 3, and the *Z*, *X*, and *Y* direction slice of the 3D reconstruction. The 5 µm coded mask was scanned with a step size of 3 µm in a linear diagonal pattern. Data were acquired at the 1BM beamline of APS.

The ESPINNet not only excels in phase reconstruction but also demonstrates remarkable capability in predicting multi-contrast images, encompassing phase, transmission, and dark-field (scattering) information. Fig. 8 ▸ illustrates the reconstructed multi-contrast images of a sample containing coal and water. The horizontal and vertical refractions in Figs. 8 ▸(*b*) and 8(*c*) are directly obtained from the neural network, while the phase in Fig. 8 ▸(*e*) is obtained through 2D integration of the angle refraction. Compared with the transmission image in (*a*), the phase image exhibits heightened contrast, facilitating a distinct demarcation between the coal and water components. The scattering image in Fig. 8 ▸(*d*) unveils the intricate structures in the sample, commonly known as the dark-field image. A comparison between the scattering and phase distributions reveals that the scattering of the coal aligns with the dark phase areas, signifying the spatial distribution of the coal. Moreover, the scattering image sharply delineates the edges of the coal, enabling a clear assessment of the fine structure density within the sample. The artifacts in the diagonal direction may be noticed in the dark field image, stemming from the diagonal linear scanning pattern employed while measuring the coal and water samples. The transmission, phase, and scattering images offer complementary insights into the test sample, and utilizing the ESPINNet enables simultaneous reconstruction of the multi-contrast images at high speed.

The rapid data acquisition and analysis facilitated by ESPINNet also prove advantageous for 3D imaging. The specimen consisting of coal and water was measured using a tomography setup with 901 projections, with each projection accompanied by only *N* = 3 speckle images. The 3D volume was reconstructed using ESPINNet and obtained using the filter back projection (FBP) method. Fig. 8 ▸(*f*) shows the phase volume and the three directional slices, revealing a clear visualization of the coal ore in the 3D slices. Benefiting from the fast data analysis speed, the whole dataset can be processed within ten minutes, while four times longer is needed for speckle tracking methods such as UMPA which is already optimized for speed. In addition, the ESPINNet allows speckle scanning positions *N* of 3 for each projection, which significantly accelerates the 3D measurements. Thanks to the high data analysis speed and the fast measurement speed, rapid 3D feedback is realized for X-ray speckle tracking imaging. Although ESPINNet has been validated on several datasets—including X-ray CRLs, bones, and coals imaged with synchrotron light sources—the sample absorption in these experiments was below 50%, as indicated by the transmission images in Figs. 7 ▸ and 8 ▸. In many industrial and biomedical scenarios involving thick specimens, absorption can exceed this level. Because the training data did not include high-absorption cases, the performance of the trained ESPINNet is likely to degrade on such samples. To enable reliable deployment in industrial settings, ESPINNet should, therefore, be retuned or further trained (*e.g.* via continued or domain-specific training) on high-absorption data.

## 4. Conclusion

A novel deep learning model, the scanning modulated pattern multi-contrast imaging network (ESPINNet), has been proposed. Trained on a simulation dataset, the ESPINNet is seamlessly applicable to various experimental datasets featuring diverse samples and modulated by both speckle and grating patterns. Compared with the XSVT and UMPA speckle tracking methods, the ESPINNet’s predictions exhibit comparable or even better accuracy. Furthermore, the ESPINNet prowess shines through its ability to generate multi-contrast images including transmission, scattering, and phase using few scanning positions, a stark improvement over the tens of scanning positions required by the scanning speckle tracking method. Benefiting from the deep learning architecture, ESPINNet accelerates the data analysis speed by a factor of four compared with the UMPA method optimized for speed and significantly reduces experimental measurement time by utilizing fewer scanning positions. This advantage positions ESPINNet as a promising candidate for real-time scanning multi-contrast imaging via modulated pattern analysis with a balanced performance in both speed and accuracy. The ESPINNet has been successfully deployed across various experimental datasets featuring different step sizes, scanning positions, scanning trajectories, speckle distributions, and grating patterns, showcasing its exceptional generalization capabilities in real data scenarios. By harnessing the capabilities of ESPINNet, high-speed 3D quantitative multi-contrast imaging encompassing transmission, scattering, and phase, at real-time speed, emerges as a feasible prospect, promising to benefit future applications in high-resolution and high-speed measurements.

## Figures and Tables

**Table 1:** Experimental parameters summary

Energy (keV)	Propagation distance (m)	Modulation	Pixel size (µm)	Sample	Scan step (µm) / trajectory	*N*
14	0.5	Mask	0.65	X-ray CRL	3 / *XY* grid	100
14	0.5	Mask	0.65	Coal–water	3 / diagonal	20
20	0.5	Checkerboard grating	0.65	Mouse bone	6 / *XY* grid	100

**Table 2:** Benchmark results of the ESPINNet

Image size (pixels)	Batch size	Time/batch (ms)	GPU memory (GB)
2048 × 2048	1	220	2.4
2048 × 2048	4	378	8.1
2048 × 2048	8	589	15.9
2048 × 2048	16	1358	31.2
1024 × 1024	1	195	0.8
1024 × 1024	4	252	2.2
1024 × 1024	8	278	4.1
1024 × 1024	16	380	7.9
512 × 512	4	208	0.7
512 × 512	8	239	1.2
512 × 512	16	250	2.1
512 × 512	32	277	4.0

## Data Availability

The datasets used and/or analyzed during the current study are available from the corresponding author on reasonable request. The code of ESPINNet is publicly available on github: https://github.com/QDLDQQiao/ESPINNet.
